# Sub-Fractions of Red Blood Cells Respond Differently to Shear Exposure Following Superoxide Treatment

**DOI:** 10.3390/biology10010047

**Published:** 2021-01-11

**Authors:** Marijke Grau, Lennart Kuck, Thomas Dietz, Wilhelm Bloch, Michael J. Simmonds

**Affiliations:** 1 Department of Molecular and Cellular Sports Medicine, German Sport University Cologne, 50933 Cologne, NRW, Germany; t.dietz@dshs-koeln.de (T.D.); w.bloch@dshs-koeln.de (W.B.); 2 Biorheology Research Laboratory, Menzies Health Institute Queensland, Griffith University, Gold Coast, QLD 4222, Australia; lennart.kuck@griffithuni.edu.au (L.K.); m.simmonds@griffith.edu.au (M.J.S.)

**Keywords:** blood rheology, nitric oxide, red blood cell deformability, shear stress conditioning, oxidative stress

## Abstract

**Simple Summary:**

Deformation of red blood cells (RBCs) is essential in order to pass through the smallest blood vessels. This cell function is impaired in the presence of high levels of free radicals and shear stress that highly exceeds the physiological range. In contrast, shear stress within the physiological range positively affects RBC function. RBCs are a heterogeneous cell population in terms of RBC age with different RBC deformability described for young and old RBCs, but whether these different sub-populations tolerate mechanical and oxidative stress to the same extent remains unknown. The results of the present investigation revealed lower RBC deformability of old RBCs compared to young RBCs and comparable reductions in RBC deformability of the sub-populations caused by free radicals. Physiological shear stress did not further affect free radical content within the RBCs and reversed the deleterious effects of free radicals on RBC deformability of old RBCs only by improving RBC deformability. The changes were aimed to be explained by changes in the formation of nitric oxide (NO), but outputs of NO generation appeared dependent on cell age. These novel findings highlight a yet less-described complex relation between shear stress, free radicals, and RBC mechanics.

**Abstract:**

Red blood cell (RBC) deformability is an essential component of microcirculatory function that appears to be enhanced by physiological shear stress, while being negatively affected by supraphysiological shears and/or free radical exposure. Given that blood contains RBCs with non-uniform physical properties, whether all cells equivalently tolerate mechanical and oxidative stresses remains poorly understood. We thus partitioned blood into old and young RBCs which were exposed to phenazine methosulfate (PMS) that generates intracellular superoxide and/or specific mechanical stress. Measured RBC deformability was lower in old compared to young RBCs. PMS increased total free radicals in both sub-populations, and RBC deformability decreased accordingly. Shear exposure did not affect reactive species in the sub-populations but reduced RBC nitric oxide synthase (NOS) activation and intriguingly increased RBC deformability in old RBCs. The co-application of PMS and shear exposure also improved cellular deformability in older cells previously exposed to reactive oxygen species (ROS), but not in younger cells. Outputs of NO generation appeared dependent on cell age; in general, stressors applied to younger RBCs tended to induce S-nitrosylation of RBC cytoskeletal proteins, while older RBCs tended to reflect markers of nitrosative stress. We thus present novel findings pertaining to the interplay of mechanical stress and redox metabolism in circulating RBC sub-populations.

## 1. Introduction

Effective microcirculatory function requires highly deformable red blood cells (RBCs), a property classically shown to be governed by the cell’s exceptional surface area-to-volume ratio, cytoplasmic viscosity being greater than the surrounding fluid, and the visco-elastic properties of the plasma membrane [1]. It is well established that free radical exposure decreases RBC deformability; extracellular reactive oxygen species (ROS) appear to induce lipid peroxidation, while intracellular ROS generation appears to cross-link membrane proteins, including hemoglobin–membrane cross-links [2]. Susceptibility of RBCs to hemolysis is consequently exacerbated, and impaired oxygen delivery to peripheral tissues may be observed [3,4,5]. The effect that shear stress exposure induces on RBCs is complex. Shear exposure within physiological limits, which has been reported to be in the range of 0.1 to 0.6 Pa within the venous system and between 1 and 15 Pa within the arterial system [6,7], appears to confer some beneficial effects and might also lead to adaptations regarding the RBC lifespan. While the regular lifespan of RBCs was reported to be around 120 days, that of the RBCs of endurance athletes is reduced to 50–70 days; this increased RBC turnover leads to a “rejuvenation” of the RBC population with improved RBC functional properties [8]. Reduced lifespan should not always be considered a physiological adaptation; however, the same is reported for some diseases such as chronic kidney failure [9], highlighting the complex determinants between RBC age and cell function. Supraphysiological levels of shear stress exposure certainly induce sublethal and/or lethal damage to RBCs. Indeed, mild shear stress exposure has even been observed to ameliorate ROS-dependent decreases in RBC deformability [10]. Furthermore, shear stress has now been consistently shown to affect RBC function in both in vitro and in vivo environments—e.g., during physical activity/exercise [8,11,12,13]. RBC deformability has been shown to be positively affected, so long as the magnitude and duration of shear stress exposure does not exceed the “subhemolytic threshold”. Blood exposure to shear stresses above this limit results in mechanical damage, thus highlighting a delicate balance between duration and magnitude of shear stress exposure, determining whether shear stress is detrimental/beneficial to RBC deformability [2,14,15]. The curious relationship between discrete shear stress exposure and enhanced RBC deformability was shown to correlate with nitric oxide (NO) availability [16,17], although the precise mechanistic relationship requires further direct evidence. Within RBCs, NO may be produced through reduction of nitrite and also via RBC-NO synthase (RBC-NOS) as a metabolic by-product of converting L-arginine to L-citrulline [18,19]. Basal NO concentration within RBCs is substantially increased via mechanical stimulation in a dose-responsive manner [20]. Increased NO bioavailability is generally beneficial for RBCs, as NO may be oxidized to nitrite and finally nitrate [21,22] and/or may transiently bind to exposed thiols within active cysteine residues in a process termed S-nitrosylation [23]. This post-translational modification has been observed in the major RBC cytoskeletal proteins α- and β-spectrin, which are pivotal in governing the flexibility of the cell membrane, and it is thought that this process contributes to the NO-dependent increase in RBC deformability [24]. On the other hand, given the abundance of O_2_ within RBCs, inevitable interactions between NO and other free radicals may result in negative outcomes. When intracellular NO reacts with superoxide, for example, formation of secondary reactive species such as peroxynitrite is well described, which detrimentally impacts RBC mechanics [25]. It is thus clear that the impact of NO on cellular mechanics of RBC is complex and dependent upon the local environment.

A challenge to understanding the impact that mechanical stress and NO production have on RBC is the fact that these cells vary substantially within a given sample. Mature circulating RBCs lack cell organelles including the nucleus, Golgi complex, and ribosomes; thus, mechanical and oxidative stresses acting on RBCs progressively cause irreparable deterioration. The native RBC population can thus be divided into a low percentage of less dense and highly flexible “young” RBCs, a high percentage of “middle age” RBCs which show the highest RBC deformability, and a low percentage of “old” RBCs that are dense and rigid [26]. It was recently shown that RBCs with distinct densities reflecting young and old cells show improved deformability following exposure to 10 Pa shear stress for 5 min [27]; those authors suspected that the different sub-populations of RBCs may have achieved this via distinct mechanisms given their vastly different basal mechanical properties. It is plausible that distinct characteristics between old and young RBCs, such as sensitivity to ROS, RBC-NOS activation levels, and S-nitrosylation of cytoskeletal proteins, might explain these earlier observations. Moreover, the effect of concomitant exposure to free radicals and mechanical stimulation on cell mechanics and NO metabolism of RBC sub-fractions is unknown but potentially valuable given the age-related differences in anti-oxidant capacity [28] and sensitivity to shear stress. The current study thus aimed to investigate potential differences in ROS-dependent alteration of RBC function between different RBC sub-populations, with a special emphasis on intracellular NO signaling.

## 2. Methods

### 2.1. Selection of Participants and Blood Sample Collection

All participants were healthy males with no known cardiovascular, respiratory, or endocrine pathologies. Participants did not report use of any medication or any history of regular cigarette smoking. The risks and benefits of the study were explained to the participants before written and witnessed consent was obtained. Anthropometric data of the participants involved in Experiment One, “impact of oxidative stress”, were as follows: 27.3 ± 4.2 years; 183.9 ± 4.2 cm; 84.2 ± 7.4 kg (*n* = 11). Anthropometric data of the participants involved in Experiment Two, “impact of shear stress following oxidative stress”, were as follows: 28.6 ± 5.2 years; 183.9 ± 6.5 cm; 84.5 ± 10.3 kg (*n* = 17).

Blood was collected from the vena mediana cubiti into sodium heparin tubes (17 IU heparin per ml blood; Becton Dickinson GmbH, Heidelberg, Germany). All experimental procedures were completed within 4 h of blood collection and each participant’s blood was used once in this study. The protocols of the present study were reviewed and approved by the Ethics Committee of the German Sports University Cologne (16.9.2013) and Griffith University (2019/808) and are consistent with the Code of Ethics of the World Medical Association (Declaration of Helsinki).

### 2.2. Experimental Protocol

The present study was conducted as a series of interconnected experiments to address the primary aims. Experiment One was designed to investigate the effect of intracellularly generated O_2_^-^ via phenazine methosulfate (PMS; Merck, Darmstadt, Germany) on the cellular deformability of young and old RBC sub-fractions. Experiment Two subsequently aimed to examine whether shear stress was able to reverse O_2_^-^-dependent deleterious effects and to examine possible differences between young and old RBC sub-populations. These measurements were complemented by studies of oxidative status and RBC-NOS-dependent S-nitrosylation of α- and β-spectrin.

#### 2.2.1. Overview of Experiment One

Whole blood was centrifuged (3500× *g* for 2 min), the buffy coat and plasma were removed, and the top and bottom 20% of RBCs were used for the following density gradient centrifugation to increase the yield of young (light) and old (dense) RBCs. RBCs were washed with 9 volumes of GASP buffer (9 mmol/L Na_2_HPO_4_, 1.3 mmol/L NaH_2_PO_4_*H_2_O, 140 mmol/L NaCl, 5.5 mmol/L glucose, and 0.8 g/L bovine serum albumin (BSA); Merck, Darmstadt, Germany). After centrifugation (3500× *g* for 4 min), the supernatant was removed, and RBCs were suspended at a ratio of 1:1 in SAH buffer (26.3 g/L BSA, 13.2 mmol/L NaCl, 4.6 mmol/L KCl, 10 mmol/L HEPES; Merck, Darmstadt, Germany). Then, 600 µl of this RBC/SAH suspension was gently transferred on top of a Percoll^®^ density gradient consisting of four layers (56%, 50%, 48%, and 46% from bottom to top), as described earlier [26]. Sub-populations of RBCs were separated according to their cell density by centrifugation (3000× *g*, 30 min). The old and young RBC sub-fractions were isolated and separately resuspended in 1% bovine serum albumin (Merck, Darmstadt, Germany) and phosphate-buffered saline (PBS; 0.1 mol/L, pH 7.4) to create a 40% hematocrit solution. Cell suspensions were subsequently incubated for 60 min in a water bath at 37 °C with gentle shaking with either 50 µmol/L PMS or PBS (as control, with the volume of PBS matching the PMS volume). Immediately following incubation, each sample was centrifuged at 1500× *g* for 5 min and washed twice with PBS. Samples were subsequently aliquoted for quantification of cell deformability, and concurrently fixed in paraformaldehyde (PFA) for immunohistochemical analysis [24]. The remaining sample was stored at −80 °C until subsequent quantification of intracellular free radical concentrations and anti-oxidative capacity or stored at −20 °C for S-nitrosylation assays.

#### 2.2.2. Overview of Experiment Two

In Experiment Two, following the O_2_^-^-generating protocol described in Experiment One, cell suspensions were then exposed to shear stress as previously described [10]. Briefly, RBCs were exposed to 5 Pa shear stress for 300 s using a cup-and-bob Couette shearing system (LORCA, Mechatronics, Hoorn, The Netherlands). Cells were inserted into a 300-µm gap between the cylinders; the outer cylinder (cup) rotated around the inner cylinder (bob) at a discrete velocity (shear rate), which was adjusted to generate the desired shear stress. Samples sheared using the cup-and-bob system were analyzed immediately following cessation of shear stress exposure for cellular deformability. Upon completion, RBCs were processed for the analysis of the study parameters as described above.

### 2.3. Oxidative Status

A commercially available assay (OxiSelect In Vitro ROS/RNS Assay Kit, Cell Biolabs Inc., San Diego, CA, USA) was employed to quantify the intracellular concentration of free reactive oxidative and nitrosative species (i.e., ROS/RNS, respectively) as described in earlier studies [29]. Briefly, a highly reactive dichlorodihydrofluorescin (DCFH) form was added to the RBC sample and concentration standards. During the 45-min incubation at room temperature, free ROS/RNS rapidly reacted with DCFH to produce the fluorescent 2’, 7’-dichlorodihydrofluorescein. The fluorescence intensity was measured with a fluorescence plate reader (Fluoroskan Ascent Microplate Fluorometer; Thermo Fisher Scientific, Dreieich, Germany) at excitation wavelength λ = 480 nm and emission wavelength λ = 530 nm and fluorescence intensity is proportional to the total ROS/RNS level within the sample.

The antioxidant capacity of the samples was determined using a commercial colorimetric assay (Abcam, Cambridge, UK) based on the reduction of copper ions (Cu^2+^) by endogenous antioxidant compounds. The reduced copper ions (Cu^+^) were chelated by a colorimetric probe which gave an absorbance peak at wavelength λ = 570 nm upon Cu^+^-binding. Top and bottom RBC sub-populations were measured against a standard of increasing Trolox concentration [30]. A total of 1 × 10^7^ RBC/mL were used for the measurements according to the manufacturer’s instructions.

### 2.4. RBC Deformability

RBC deformability was measured using a laser diffraction ektacytometer (LORCA, Mechatronics, Hoorn, the Netherlands) [31]. RBCs were diluted at 1:250 in polyvinylpyrrolidone (PVP) (30 ± 0.5 mPa·s; 7.4 ± 0.5 pH; 290 ± 5 mOsm·kg^−1^) and the RBC/PVP suspension was inserted into the gap between the two cylinders (i.e., cup and bob). A low-power laser (670 nm; <5 mW) located within the inner cylinder was projected through the blood suspension, generating a diffraction pattern that reflects the average cell morphology in the sample. The diffraction patterns were analyzed in real time to determine cell deformation. RBCs were sheared at ten discrete shear stresses over the range 0.3–50.0 Pa, such that an ellipse could be fit to the resultant laser diffraction patterns. An elongation index (EI) was calculated at each shear stress using the long and short axes of the ellipse (a and b, respectively) using the equation: EI = (a − b)/(a + b) [32].

### 2.5. Immunohistochemical Staining

RBCs were fixed in 4% PFA as described earlier [11,24] and blood smears were prepared. A test and a control area were marked on each slide and RBCs were washed with Tris-buffered saline (0.1 mol·L^−1^, pH 7.6) twice before being incubated with 0.1% Trypsin solution for 30 min at 37 °C to permeabilize the cell membrane. Cells were then incubated in solution (2% hydrogen peroxide; 80% methanol) and the backgrounds of the samples were blocked using 3% skim milk in Tris-buffered saline (TBS) to minimize non-specific binding. The test field of each slide was incubated with the respective primary antibody rabbit anti-human NOS III Serine1177 (1:150, Merck, Darmstadt, Germany) or Anti-Nitrotyrosine (dilution 1:500, Upstate, Millipore-Merck, Darmstadt, Germany). The control field on each slide was treated identically, except that it was not incubated with the primary antibody during this step. Unbound primary antibody was then washed off the slides prior to blocking with 3% normal goat serum. Cells were then incubated with a secondary antibody (goat anti-rabbit; dilution: 1:400, Dako, Glostrup, Denmark) and staining was developed using 3,3-diaminobenzidine-tetrahydrochloride (Sigma, St. Louis, MO, USA). Slides were then dehydrated by exposure to alcohol solutions of increasing concentrations and sealed using a purpose-mounting medium. Images of the cells were taken using an Axiophot 1 microscope (Zeiss, Oberkochen, Germany) coupled to a camera (Progres Gryphax Prokyon; Jenoptik Optical Systems GmbH, Jena, Germany) with a magnification of 400-fold and were analyzed with the software ImageJ (National Institutes of Health, Bethesda, MD, USA). For staining intensity analysis, mean grey values were measured and total immunostaining intensity was calculated as previously described [24].

### 2.6. S-Nitrosylation Assay

The S-Nitrosylated Protein Detection Assay Kit (Cayman Chemicals, Ann Arbor, USA) was used and involved the following steps: (1) RBC lysis and blocking of free thiol (SH) groups; (2) cleavage of S-NO bonds present in the sample; and (3) biotinylation of SH groups. Protein concentration of the samples was determined using the DC-Protein Assay Kit (BioRad, Munich, Germany) and 50 µg protein was loaded into the lanes of a 3–8% Tris-acetate gel (BioRad, Munich, Germany). Proteins were separated for 1 h with constant 90 mA according to their charge and mass in a 1 x XT Tricine running buffer (BioRad, Munich, Germany). Separated proteins were transferred to a polyvinylidene difluoride membrane (0.45-mm pore size) and the membrane was blocked in 2% BSA in TBS containing 0.1% Tween^®^ 20 prior to incubation with horseradish peroxidase (dilution 1:2000). The immunoreactive bands were developed using an enhanced chemiluminescence kit (Thermo Scientific, Darmstadt, Germany) and band intensities were calculated in ImageJ software (National Institutes of Health, Bethesda, USA) (adapted to [24]).

### 2.7. Statistical Analysis

All results are presented as mean ± standard error (SE). Normal distribution of the data was calculated, and a one-way analysis of variance with Tukey’s post-hoc test was applied to compare sub-population intervention effects. Statistical analyses were conducted using commercial software (Prism 6.0, GraphPad Software Inc., La Jolla, CA, USA) with an alpha level of 0.05 to determine statistically significant effects. Effect size (ES) was also calculated as Cohen’s D for selected parameters. Thus, an ES of 0.2 is considered to display a small effect, ES 0.4 represents a medium effect, and ES 0.8 represents a large effect.

## 3. Results

### 3.1. Oxidative Status

**Figure 1:**
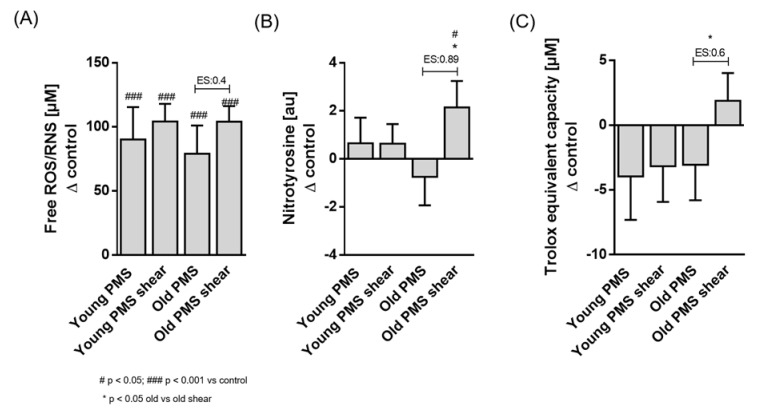
Markers of oxidative stress in old and young red blood cells (RBCs) after phenazine methosulfate (PMS) and/or shear stress application relative to baseline. (**A**) Free reactive oxidative and nitrosative species (ROS/RNS) levels were significantly higher in PMS-treated samples compared to the control condition (*p* < 0.001) but comparable between young and old RBCs. Shear stress application additionally increased ROS/RNS levels in the old PMS-treated RBCs (effect size (ES) = 0.4). (**B**) Nitrotyrosine staining was not affected by PMS nor by shear stress in young RBCs, but the combination of shear stress and PMS application significantly increased nitrotyrosine signal in old RBCs compared to control and compared to non-sheared samples (*p* < 0.05). (**C**) Antioxidant capacity, reflected by Trolox equivalent capacity, was lower in PMS-treated young and old RBCs. Shear stress significantly increased antioxidant capacity of old PMS-treated RBCs (*p* < 0.05).

The concentration of total free radical species within RBCs significantly increased (*p* < 0.001, Figure 1A) following PMS treatment, irrespective of cell age. Moreover, application of shear stress to PMS-treated old RBCs resulted in a further increase in cytosolic ROS/RNS (ES = 0.4, *p* < 0.05), while no significant effect of shear stress was observed in young RBCs. 

Basal levels of nitrated tyrosine residues, indicative of peroxynitrite (ONOO^-^) generation, were not significantly altered by PMS treatment. Exposure of old RBC sub-fractions to shear stress, however, significantly increased nitrotyrosine staining (ES = 0.89, *p* < 0.05; Figure 1B), while shear stress exposure had no effect on young RBCs.

Total antioxidant capacity (TAC) was significantly lower in old RBC sub-populations compared to young RBCs (42.45 ± 12.65 a.u. vs. 31.78 ± 7.09 a.u.; *p* < 0.05). PMS treatment resulted in mildly decreased TACs of young and old RBCs, although this was not statistically significant. Exposure of PMS-treated old sub-fractions to shear stress significantly increased TAC compared to old non-sheared cells (ES = 0.6, *p* < 0.05; Figure 1C), while no effect was observed in young RBC fractions.

### 3.2. RBC Deformability

**Figure 2:**
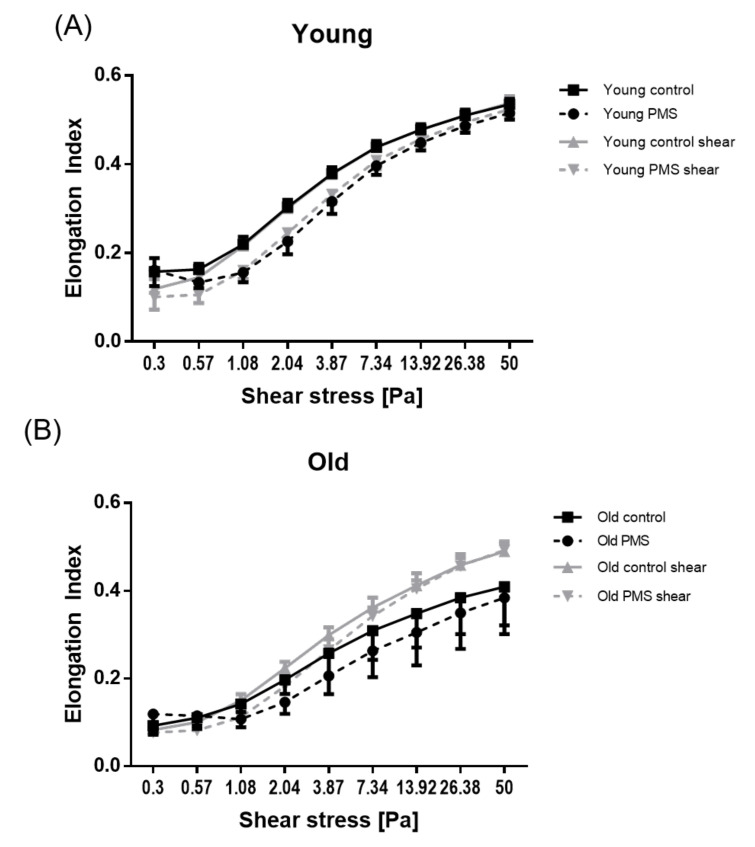
RBC deformability responses to PMS and prolonged shear stress exposure. RBC deformability of young RBCs was significantly higher than of old RBCs. (**A**) Deformability of young RBC was significantly reduced after PMS incubation but was not affected by shear stress. (**B**) Deformability of old RBCs was significantly reduced by PMS, but increased after shear stress application; the combination of both PMS and shear stress also led to an increase in RBC deformability.

A clear difference was observed in RBC deformability for “untreated” young and old sub-populations; cellular deformability was significantly decreased in older cells (*p* < 0.001). The response of each sub-population to PMS and/or mechanical stimulation was also distinct. Young RBCs incubated with PMS had significantly decreased cellular deformability (*p* < 0.001; not shown in graph). Furthermore, application of 300 s of shear stress exposure to young RBCs had no effect on cellular deformability (Figure 2A). Old RBCs showed significantly reduced RBC deformability after PMS incubation for shear stresses of 0.3 and 1.08–50 Pa (*p* < 0.001). Shear stress exposure for 300 s significantly increased the RBC deformability of old RBCs (*p* < 0.05 for measured shear stresses of 2.04–13.92 Pa and *p* < 0.01 for measured shear stresses of 26.38 and 50 Pa). This observation was maintained even in older RBCs treated with PMS (*p* < 0.01 for measured shear stresses of 0.3, 0.57, and 2.04 Pa and *p* < 0.001 for measured shear stresses of 3.87–50 Pa; Figure 2B).

### 3.3. RBC-NOS Activation

**Figure 3:**
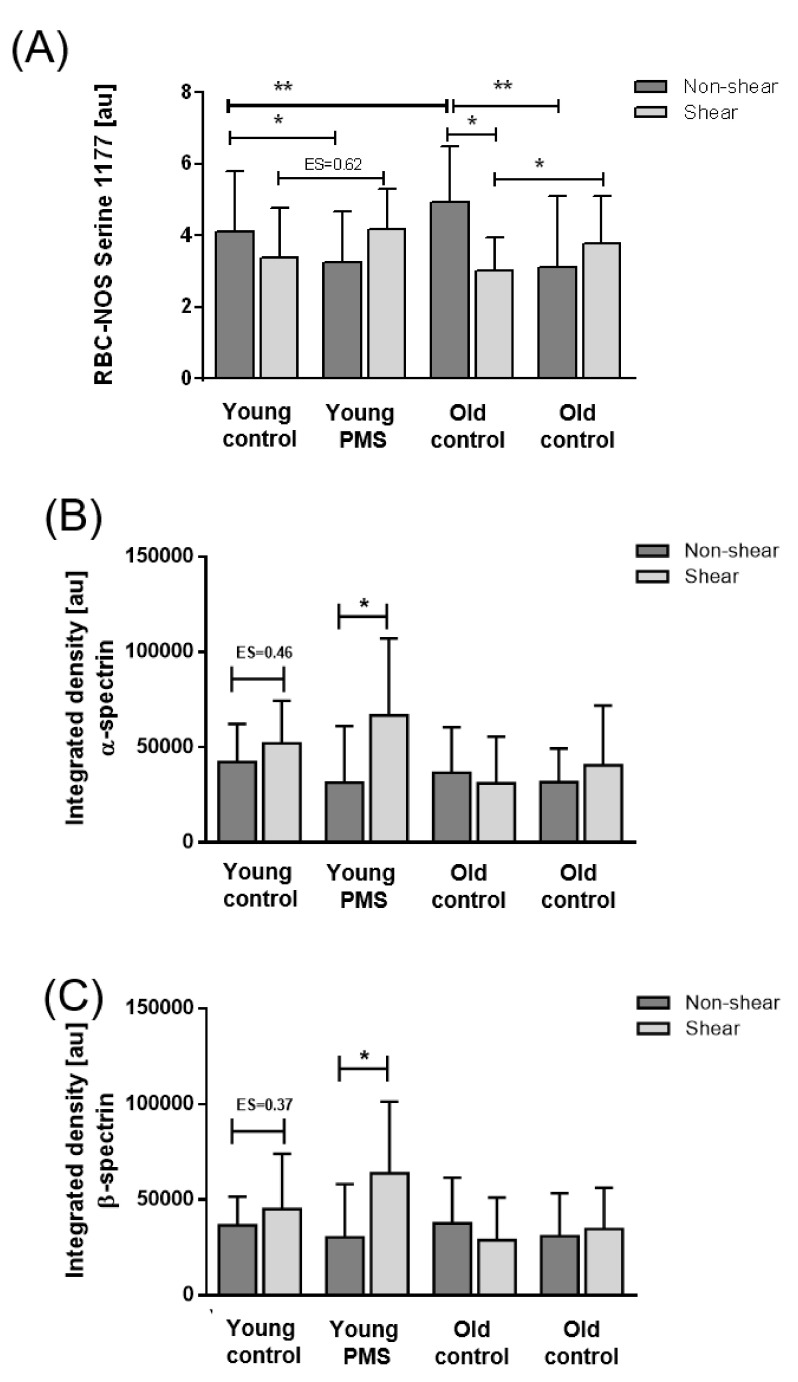
RBC nitric oxide synthase (RBC-NOS) activation and S-nitrosylation of the cytoskeletal spectrins in the RBC sub-fractions after exposure to PMS and prolonged shear stress. (**A**) RBC-NOS activation, reflected by serine 1177 phosphorylation, significantly decreased in young RBCs after PMS incubation (*p* < 0.05). Shear stress application following PMS incubation increased RBC-NOS activation in young RBCs (ES = 0.62). In old RBCs, RBC-NOS activation decreased after PMS application but also after shear stress exposure. Shear stress following PMS application led to a significant increase in RBC-NOS activation when compared to shear stress condition solely. S-nitrosylation of (**B**) α-spectrin and (**C**) β-spectrin increased in young control and PMS-treated RBCs after shear stress exposure while no such an effect was observed in old RBCs.

Basal RBC-NOS activation, reflected by phosphorylation at ser1177 (p1177), was increased in old RBC sub-fractions compared to young RBC fractions (*p* < 0.01). Treatment with PMS significantly decreased RBC-NOS activation in young fractions (*p* < 0.05) and co-application of PMS and shear stress enhanced RBC-NOS activation signals in young RBCs (ES: 0.62). PMS also decreased p1177 in old RBC sub-populations (*p* < 0.05). Exposure of old RBCs to shear stress significantly decreased p1177 signaling (*p* < 0.01). Co-application of PMS and shear stress enhanced RBC-NOS activation signals in old RBCs (*p* < 0.05) when compared to control conditions (Figure 3A).

### 3.4. S-Nitrosylation of Cytoskeletal Proteins

Basal S-nitrosylation of both α- and β-spectrin was comparable between young and old RBCs. Following shear stress exposure, however, S-nitrosylation of α- and β-spectrin increased in young control RBC fractions (ES = 0.46 and ES = 0.37, respectively). Moreover, young RBC sub-populations previously exposed to PMS also exhibited an increase in S-nitrosylated spectrin (*p* < 0.05) with shear stress. Neither stimulation with shear stress nor PMS treatment elicited significant alterations in S-nitrosylated spectrin of old RBC sub-populations (Figure 3B,C).

## 4. Discussion

The salient results of the current study indicate that RBC sub-populations at either end of the in vivo age spectrum show similar responses to PMS-induced superoxide anion-dependent ROS generation but different responses to moderate shear stress application.

RBCs are a heterogenous cell population in terms of cell age. While around 10–15% of circulating RBCs reflect newly formed and thus “young” cells, another 10–15% of the cells reflect physical properties that may be characterized as “old”. The remaining 70–80% of circulating cells are considered middle-aged cells [26]. Extracellular ROS released from neutrophils, macrophages, and endothelial cells [3] are taken up by the RBCs in the microcirculation but are also generated within the RBCs during auto-oxidation of hemoglobin and possibly by NADPH oxidases [3,33]. These processes lead to increased accumulation of reactive species during cell aging [34], which is consistent with our observation that older RBCs had greater levels of ROS/RNS compared with young RBCs [35]. In parallel, levels of anti-oxidants, such as reduced glutathione, and activity of anti-oxidative enzymes may decrease during cell aging, although the exact relation during RBC senescence remains poorly understood [28]. It was presently observed that shear stress exposure increased the total free radicals in older RBCs. A similar observation has been made in other cell lines, including endothelial cells [36]. It appears plausible that shear stress-dependent processes that increase NO production may promote secondary free radicals in certain environments, given that NO readily reacts with superoxide anion to form peroxynitrite and thus promotes nitration of tyrosine residues [37]. Indeed, in the present study, RBC nitrotyrosine significantly increased in PMS-treated old RBCs following shear stress exposure, although this was not observed in young RBCs. It is plausible that this response may be explained by the lower total antioxidant capacity in older RBCs compared to young RBCs. It indeed appears that during senescence, RBCs lose some of their capacity to quench free radicals [28].

RBC deformability was significantly decreased following PMS incubation, supporting earlier observations [3,4,10]. The present study extends these observations by demonstrating that a similar magnitude of effect was induced in both young and old RBCs, even though older RBCs clearly exhibit reduced cellular deformability in absolute terms prior to treatment [26,27,38]. During in vivo aging, RBCs exhibit decreased metabolic activity and morphological changes, including a reduction in cell volume, altered cell shape, and imbalances in surface chemistry [39]. These processes are believed to precipitate decreased membrane fluidity, reduced sialic acid content and CD47, and externalization of phosphatidylserine [40]. Furthermore, the aging process may lead to heightened susceptibility to oxidative stress, with imbalances in ROS and RNS due to reactions involving hemoglobin appearing central [41]. Furthermore, constriction of microvesicles that form during the aging process leads to the release of microparticles in an attempt to remove oxidative waste products, although this also corresponds with cell shrinkage and blebbing of the membrane [42]. The altered surface-to-volume ratio as a result of the vesiculation has a direct impact on reducing cellular deformation [39,43,44]. It is thus curious that it was recently reported that mild shear stress exposure ameliorated the ROS-dependent impairment of RBC deformability [5], which was also observed herein. This unusual observation highlights the multifaceted nature of cell mechanics in RBCs. In the present study, shear stress exposure increased cell deformability in old cells even following PMS treatment, whereas young RBCs retained their already superior cellular deformability following shear stress exposure. The rationale for this divergent observation may relate to the fact that older RBCs, with reduced basal cellular mechanics, may have benefited from mild shear stress conditioning; indeed, the elevated ROS/RNS and nitrotyrosine levels in older RBCs following combined PMS incubation and shear stress application may indicate a complex free radical environment that led to benefits regarding cellular deformability. The increased TAC which was observed in parallel might be involved in this finding. A simple interpretation is also plausible—that the benefits conferred by mild shear stress exposure may have been insufficient to improve the already superior basal cell mechanics of young RBCs. The distinct responses of RBC sub-populations to shear stress are in contrast to earlier work reporting that the most dense and the least dense RBCs each respond favorably to shear exposure [27]. Tomschi and colleagues reported no change in RBC deformability of different sub-fractions after exercise [45], although the beneficial effects of shear stress on RBC deformability appear transient and, thus, may be missed if measured >5 min following the intervention [46]. Distinct responses to shear exposure among the various sub-fractions of RBCs might also be explained by different approaches to shear stress application. While McNamee and colleagues [27] applied 10 Pa for 5 min, the shear stress applied herein was 5 Pa for 5 min; it is thus plausible that old and young cells have different sensitivities to shear stress, where younger cells were not sufficiently stimulated by 5 Pa shear stress in the present study and rather require ~10 Pa. Furthermore, RBC deformability appears to be impacted by RBC-NOS activation [19,24], and the present data tend to confirm previous findings that older cells had higher RBC-NOS activation compared with young cells [26]. Those authors reported that the NO content of older RBCs was higher and plausibly presented a compensatory mechanism to maintain RBC deformability [26].

In the present study, shear exposure did not affect RBC-NOS activation in young or old cells. It is known that the duration and intensity of a shear stress stimulus are important determinants of RBC-NOS activation [11,14,47,48,49], although the present data indicate that the extreme sub-populations of young/old cells may have different sensitivities to shear stress compared with whole blood with a large number of middle-aged cells. It was intriguing that shear stress exposure had no significant effect on RBC-NOS activation in old RBCs, yet RBC deformability significantly increased for the same samples following shear stress. This observation is unique in that increased RBC deformability following shear stress is almost universally reported to correlate with increased RBC-NOS activation, thus suggesting that shear stress conditioning may augment cell mechanics independently of RBC-NOS activation. Similar observations were made after an acute bout of exercise in sickle cell anemia patients, which appeared to confound the authors [50]. It thus seems that the relationships between RBC-NOS, NO, and RBC deformability are complex and have likely been oversimplified in contemporary works. A present example is the unexpected observation that combined mechanical and oxidative stress led to increased RBC-NOS activation, which is in stark contrast to the conventional wisdom that superoxide negatively affects both cell mechanics and RBC-NOS activation. Further evaluation of these interactions should yield greater understanding of the mechanisms dictating negative/positive responses.

Prior works have reported that the primary mechanism for enhanced RBC deformability following RBC-NOS activation is associated S-nitrosylation of the RBC cytoskeleton, most likely α- and β-spectrin [24]. In the present study, S-nitrosylation of spectrin did not differ between the sub-populations of RBCs, suggesting that reduced deformability of older RBCs may be less affected by changes in post-translational S-nitrosylation of cytoskeletal spectrins. Furthermore, S-nitrosylation remained unaltered following PMS treatment, also providing evidence that superoxide exerts its effect on cell mechanics independent of spectrin. Combined application of shear stress and superoxide-generating PMS increased S-nitrosylation in young RBCs but not in old RBCs, which is unexpected given that this combination would be expected to generate secondary nitrogen species that would result in greater nitrotyrosine (as indicated in Figure 1). It thus appears that the target of intracellularly generated NO is highly dependent on a complex milieu of intracellular processes.

## 5. Conclusions

In summary, the present results highlight a complex relation between shear stress, free radicals, and RBC mechanics, which appear to have been oversimplified in prior works. The present investigation indicates that cell density and, thus, age have a profound effect on the cell mechanics and the underlying biochemical processes within RBCs, thus prompting more questions on the regulation of the physical properties of blood. Specifically, these works indicate that improved RBC deformability following shear stress may not only depend on RBC-NOS/S-nitrosylation of the cytoskeleton, and shifts focus toward the redox status of these vital cells. Future studies will extend these findings to examine different cell functions, with a specific focus on the different metabolic and anti-oxidative capacities of discrete RBC sub-populations; this information will provide new perspectives on diseases involving altered oxidative stress, cell rigidity, and abnormal NO availability. Furthermore, integration of the present assays into microfluidic models may provide tantalizing opportunities for higher fidelity assessments.

## Data Availability

The data presented in this study are available on request from the corresponding author. The data are not publicly available due to ethical reasons.
